# Sepsis-related coagulopathy treatment based on the disseminated intravascular coagulation diagnostic criteria: a post-hoc analysis of a prospective multicenter observational study

**DOI:** 10.1186/s40560-023-00656-5

**Published:** 2023-03-05

**Authors:** Takeshi Wada, Kazuma Yamakawa, Daijiro Kabata, Toshikazu Abe, Seitaro Fujishima, Shigeki Kushimoto, Toshihiko Mayumi, Hiroshi Ogura, Daizoh Saitoh, Atsushi Shiraishi, Yasuhiro Otomo, Satoshi Gando, the JAAM FORECAST Group, Junichi Sasaki, Joji Kotani, Naoshi Takeyama, Ryosuke Tsuruta, Kiyotsugu Takuma, Norio Yamashita, Shin-ichiro Shiraishi, Hiroto Ikeda, Yasukazu Shiino, Takehiko Tarui, Takaaki Nakada, Toru Hifumi, Kohji Okamoto, Yuichiro Sakamoto, Akiyoshi Hagiwara, Tomohiko Masuno, Masashi Ueyama, Satoshi Fujimi, Yutaka Umemura, Yasumitsu Mizobata, Yasuo Yamada, Satoru Sugiyama, Hiroshi Ishida, Eichi Narimatsu, Koji Miyasho, Toshio Kanai, Satoru Miyatake, Ryouji Iiduka, Hiroshi Imamura, Yasuaki Mizushima, Yoshitake Sato, Manabu Nemoto, Hiroyuki Hanada, Yasuo Shichinohe, Kenji Hirahara, Akihide Kon, Manabu Sugita, Yasutaka Naoe, Manabu Kirita, Daikai Sadamitsu, Masahiro Yoshida

**Affiliations:** 1 Division of Acute and Critical Care Medicine, Department of Anesthesiology and Critical Care Medicine, Hokkaido University Faculty of Medicine, N15, W7, Kita-Ku, Sapporo, Japan; 2 Department of Emergency Medicine, Osaka Medical and Pharmaceutical University, Takatsuki, Japan; 3 Department of Medical Statistics, Graduate School of Medicine, Osaka Metropolitan University, Osaka, Japan; 4 Department of Emergency and Critical Care Medicine, Tsukuba Memorial Hospital, Tsukuba, Japan; 5 Health Services Research and Development Center, University of Tsukuba, Tsukuba, Japan; 6 Center for General Medicine Education, Keio University School of Medicine, Tokyo, Japan; 7 Division of Emergency and Critical Care Medicine, Tohoku University Graduate School of Medicine, Sendai, Japan; 8 Department of Trauma, Critical Care Medicine and Burn Center, Community Healthcare Organization, Chukyo Hospital, Nagoya, Japan; 9 Department of Traumatology and Acute Critical Medicine, Osaka University Graduate School of Medicine, Suita, Japan; 10 Division of Traumatology, Research Institute, National Defense Medical College, Tokorozawa, Japan; 11 Emergency and Trauma Center, Kameda Medical Center, Kamogawa, Japan; 12 Trauma and Acute Critical Care Center, Medical Hospital, Tokyo Medical and Dental University, Tokyo, Japan; 13 Department of Acute and Critical Care Medicine, Sapporo Higashi Tokushukai Hospital, Sapporo, Japan

**Keywords:** Anticoagulant therapy, Disseminated intravascular coagulation, Multiple organ dysfunction syndrome, Prothrombin time, Sepsis

## Abstract

**Background:**

The development of disseminated intravascular coagulation (DIC) in patients with sepsis has been repeatedly confirmed as a factor associated with poor prognosis. Anticoagulant therapy has been expected to improve sepsis patient outcomes, whereas no randomized controlled trials have demonstrated the survival benefit of anticoagulant therapies in non-specific overall sepsis. Patient selection based on the component of “high disease severity” in addition to “sepsis with DIC” has recently proved important in identifying appropriate targets for anticoagulant therapy. The aims of this study were to characterize “severe” sepsis DIC patients and to identify the patient population benefiting from anticoagulant therapy.

**Methods:**

This retrospective sub-analysis of a prospective multicenter study included 1,178 adult patients with severe sepsis from 59 intensive care units in Japan from January 2016 to March 2017. We examined the association of patient outcomes, including organ dysfunction and in-hospital mortality, with the DIC score and prothrombin time-international normalized ratio (PT-INR), one of the components of the DIC score, using multivariable regression models including the cross-product term between these indicators. Multivariate Cox proportional hazard regression analysis with non-linear restricted cubic spline including a three-way interaction term (anticoagulant therapy × the DIC score × PT-INR) was also performed. Anticoagulant therapy was defined as the administration of antithrombin, recombinant human thrombomodulin, or their combination.

**Results:**

In total, we analyzed 1013 patients. The regression model showed that organ dysfunction and in-hospital mortality deteriorated with higher PT-INR values in the range of < 1.5 and that this trend was more pronounced with higher DIC scores. Three-way interaction analysis demonstrated that anticoagulant therapy was associated with better survival outcome in patients with a high DIC score and high PT-INR. Furthermore, we identified a DIC score ≥ 5 and PT-INR ≥ 1.5 as the clinical threshold for identification of optimal targets for anticoagulant therapy.

**Conclusions:**

The combined use of the DIC score and PT-INR helps in selecting the optimal patient population for anticoagulant therapy in sepsis-induced DIC. The results obtained from this study will provide valuable information regarding the study design of randomized controlled trials examining the effects of anticoagulant therapy for sepsis.

*Trial registration*: UMIN-CTR, UMIN000019742. Registered on November 16, 2015.

**Supplementary Information:**

The online version contains supplementary material available at 10.1186/s40560-023-00656-5.

## Background

The Analysis for the Global Burden of Disease demonstrated the incidence of sepsis to be 48.9 million and the number of sepsis-related deaths to be 11 million worldwide in 2017 [1]. Thus, 20% of worldwide deaths resulted from sepsis, which is greater than the proportion of deaths from cancer. The Third International Consensus Definitions for Sepsis and Septic Shock (Sepsis-3) define sepsis as “life-threatening organ dysfunction caused by a dysregulated host response to infection” [2], and disseminated intravascular coagulation (DIC) is one of the most common causes of organ dysfunction associated with sepsis [3]. DIC is characterized by systemic hypercoagulation followed by microcirculatory endothelial dysfunction, leading to the development of organ dysfunction and adversely affecting patient outcomes [3–6]. Two large-scale observational studies have clearly reported significantly higher mortality rate among sepsis patients with DIC than among those without DIC [7, 8]. In addition, a nationwide multicenter observational study suggested that the screening and diagnosis of DIC were associated with a survival benefit in patients with sepsis [9], implying that some interventions for coagulation disorders associated with sepsis may contribute to improved patient outcomes.

However, no large-scale randomized controlled trials (RCTs) have demonstrated the survival benefit of anticoagulant therapies for patients with sepsis. However, a meta-analysis has indicated the important concept regarding anticoagulant therapy for sepsis; the optimal population benefiting from anticoagulant therapy consists of patients with sepsis and DIC, rather than those with sepsis only [10]. In addition, a recent study proposed that patient selection for anticoagulant therapy should be based on disease severity as indicated by the Sequential Organ Failure Assessment (SOFA) or Acute Physiology and Chronic Health Evaluation (APACHE) II scores, in addition to “sepsis with DIC” [11].

The Japanese Association for Acute Medicine (JAAM) DIC diagnostic criteria [12] (see Additional file 1: Table S1) as well as the International Society on Thrombosis and Haemostasis (ISTH) overt DIC diagnostic criteria [4] account for the most widely used diagnostic criteria [13]. The JAAM scoring system has been repeatedly validated as a highly sensitive and simple diagnostic method [7, 13, 14], consisting of systemic inflammatory response syndrome (SIRS) criteria and global coagulation markers including prothrombin time (PT) ratio, platelet counts, and fibrin/fibrinogen degradation products [12]. The aforementioned SOFA score has also been found to be useful in identifying the optimal patient population benefiting from anticoagulant therapy in patients with sepsis. However, the limitations of this score, including discrepancies with current clinical practices, such as the effect of dopamine in scoring circulatory system, lack of positive end-expiratory pressure in scoring respiratory system, and determination of cut-off values based on expert opinion rather than statistical methods, have been noted [15]. Previous studies have demonstrated that PT values are strongly correlated with SOFA scores [16] and that mortality in patients with sepsis increases with an increase in the PT value [17], further indicating that PT values could serve as an alternative to the SOFA score.

From this perspective, this study aimed to characterize severely septic DIC patients using the DIC score and PT value and to identify the patient population that would best benefit from anticoagulant therapy based on these two indicators.

## Methods

### Study design, setting, and ethical approval

This study was a retrospective sub-analysis of a cohort of patients with sepsis in the JAAM Focused Outcomes Research in Emergency Care in Acute Respiratory Distress Syndrome, Sepsis, and Trauma (FORECAST) database. The main investigation in this cohort had evaluated the characteristics, management, and outcomes among patients with severe sepsis in Japan [18]. The JAAM FORECAST study was a multicenter prospective study of acutely ill patients, including those with acute respiratory distress syndrome, sepsis, and trauma, that collected consecutive samples from 59 intensive care units in Japan from January 2016 to March 2017. This manuscript was written in accordance with the STROBE reporting guidelines (https://www.strobe-statement.org/).

This study was approved by the JAAM and the Ethics Committee of all participating hospitals, waiving written informed consent (JAAM, 2014-01, Hokkaido University Graduate School of Medicine, Head institute of the FORECAST group, 014-0307) since these were already previously obtained from each patient or their next of kin. Furthermore, the study was performed in accordance with the tenets underlying the Declaration of Helsinki.

### Participants

The sepsis sub-cohort of the JAAM FORECAST study included adult patients aged ≥ 16 years with severe sepsis and septic shock according to the Sepsis-2 criteria [19] who had been admitted to the intensive care unit. The exclusion criteria were as follows: end-of-life care or resuscitated status after cardiac arrest at the time of sepsis diagnosis. The current study also excluded patients with substantial missing data, such as DIC scores at study enrollment. The size of the study population was dependent on the study period. All patients were followed up until discharge.

Participants were divided into four groups according to a PT-international normalized ratio (INR) ≤ 1.2, 1.2 < PT-INR ≤ 1.4, 1.4 < PT-INR ≤ 1.6, and 1.6 < PT-INR, based on existing diagnostic scoring systems of sepsis-related coagulopathies [12, 17, 20].

### Definitions

SIRS, sepsis, severe sepsis, and septic shock were defined according to the American College of Chest Physicians/Society of Critical Care Medicine consensus conference (Sepsis-1) [21] and its revised version (Sepsis-2) [19]. Moreover, disease severity was assessed based on the APACHE II score [22], and organ dysfunction was evaluated based on the SOFA score [23]. Multiple organ dysfunction syndrome (MODS) was defined as a SOFA score of ≥ 12 based on a previous study [23]. Baseline comorbidities were assessed by the Charlson Comorbidity Index (CCI) [24]. Additionally, DIC was diagnosed based on the JAAM DIC scoring system using PT-INR as a substitute for the PT ratio [12]. Anticoagulant therapy was defined as the administration of antithrombin, rhTM, or their combination based on the J-SSCG 2020 [25]. Serine protease inhibitors mentioned in this guideline were not included in anticoagulant therapy based on the results of a previous study reporting that their administration as treatment for sepsis-induced DIC had decreased over the years in Japan [26]. Heparin was also excluded from the anticoagulant therapy since no corresponding data for sepsis were present in the JAAM FORECAST sepsis database. There were no pre-determined, definitive indications for anticoagulant therapy, and anticoagulants were administered at the discretion of the attending physicians based on the treatment policies of each hospital. The standard dosage and administration of antithrombin for sepsis-induced DIC in Japan is 1500 U/day or 30 U/kg/day for 3–5 days, whereas that of rhTM is 380 U/kg for 6 days.

### Data collection

Data were collected from the electronic data capture system, which was compiled by the FORECAST investigators. Patient information included baseline characteristics, various comorbidities, activity of daily living (ADL), suspected sites of infection, indicators of severity associated with sepsis, and therapeutic interventions. The primary outcome was in-hospital all-cause mortality. The SOFA scores and MODS prevalence at 72 h after admission were recorded as secondary outcomes.

### Statistical analyses

Descriptive statistics used numbers for categorical variables and median values (interquartile range) for continuous variables. Categorical variables were compared using Chi-squared tests or Fisher’s exact test, as appropriate. The Mann–Whitney *U* and Chi-squared tests were used to determine the differences between two groups. To compare results among multiple groups, Kruskal–Wallis one-way analysis was adopted. We examined the association of prognosis with the DIC score and PT-INR value, which were assessed at the time of admission, using multivariable regression models including the cross-product term between the DIC score and PT-INR value.

To estimate the occurrence of MODS at 72 h after admission, we used multivariable logistic regression models with adjustment for age, sex, CCI, and ADL at the time of admission. We allowed the non-linear association of PT-INR with the outcome variable through a restricted-cubic-spline with knot three. Furthermore, we estimated the correlation of the SOFA score at 72 h after admission with the time to in-hospital mortality within 90 days of admission using a multivariable non-linear regression model and multivariable Cox proportional hazard regression model, respectively. In these models, the consideration of the non-linear association of PT-INR values and adjustment for the covariates were conducted similarly to the logistic regression model described above. Furthermore, to illustrate the effect of the anticoagulant therapy according to the DIC score and PT-INR value, we performed similar analyses as described above, including three- and two-way cross-product terms between the presence or absence of anticoagulant therapy, DIC scores, and PT-INR values. In the regression models, all missing values were imputed using multiple imputation methods with the predictive mean matching approach with five repetitions. We compared disease severity using the SOFA score at 0 h and APACHE II score, as well as in-hospital mortality between the groups with and without anticoagulant therapy using several DIC score and PT-INR value combinations as a sensitive analysis.

All statistical hypothesis tests were performed with a two-sided 5% significance level using SPSS version 26 (IBM Japan, Tokyo, Japan) and R version 4.1.1 (https://cran.r-project.org/).

## Results

### Patient demographics and baseline characteristics

A total of 1,184 consecutive patients with severe sepsis fulfilling the inclusion criteria were included in the JAAM FORECAST sepsis study. As shown in Additional file 2: Fig. S1, six patients with missing values exceeding the threshold (> 170) detected by a one-sample robust regression with M estimator were excluded. In addition, 165 patients were excluded as shown in Table 1 and Additional file 1: Tables S2 and S3 due to missing information regarding the DIC score or PT-INR value at day 0. In total, 1013 patients were analyzed in this study. Table 1 shows baseline clinical and demographic characteristics of the patients divided into four groups according to the PT-INR value. No significant differences were observed in patient characteristics, including age, sex, and preexisting conditions such as CCI and ADL, among the groups. The SOFA and DIC scores at 0 h increased with higher PT-INR values, then plateaued at PT-INR values > 1.4. The most common site of infection was the lungs (31.3%), followed by the abdomen (26.8%), urinary tract (18.6%), and skin/soft tissues (9.7%). In addition, the proportion of patients receiving various therapeutic interventions generally tended to increase with increasing PT-INR values up to 1.6. The proportion of patients with PT-INR values > 1.6 receiving AT or rhTM was lower than that of patients with PT-INR values between 1.4 and 1.6. A comparison of platelet counts, global markers of coagulation and fibrinolysis, and clinical outcomes are shown in Additional file 1: Tables S2 and S3.

**Table 1:** Baseline characteristics in sepsis patients according to the PT-INR value

	Overall (*N* = 1,013)	PT-INR ≤ 1.2 (*N* = 505)	1.2 < PT-INR ≤ 1.4 (*N* = 265)	1.4 < PT-INR ≤ 1.6 (*N* = 106)	1.6 < PT-INR (*N* = 137)	*P*-value
Patient characteristics						
Age, years	72 (63–81)	74 (64–82)	70 (63–79)	72 (62–-82)	72 (62–79)	0.182
Sex (female/male)	391/622	198/307	100/165	38/68	55/82	0.887
Preexisting conditions						
Charlson Comorbidity Index	1 (0–2)	1 (0–2)	1 (0–2)	1 (0–3)	2 (0–3)	0.070
ADL dependent/independent	223/779	119/386	63/201	17/89	34/103	0.403
Disease severity						
APACHE II score	22 (17–29)	21 (16–28)	24 (17–31)^a^	24 (18–33)^a^	28 (18–33)^a,b^	< 0.001
SOFA score	9 (6–11)	8 (5–10)	9 (6–12)^a^	11 (7–13)^a,b^	11 (7–13)^a,b^	< 0.001
SIRS score	3 (2–4)	3 (2–4)	3 (3–4)	3 (3–4)	3 (2–3)	0.081
DIC score	4 (2–5)	3 (2–5)	4 (3–6)^a^	5 (3–6)^a,b^	5 (2–6)^a^	< 0.001
DIC, % (freq.)	50.9 (516)	40.4 (204)	59.2 (157)^a^	70.8 (75)^a,b^	58.4 (80)^a^	< 0.001
Septic shock, % (freq.)	62.6 (634)	55.2 (279)	64.9 (172)^a^	78.3 (83)^a,b^	73.0 (100)^a^	< 0.001
Blood culture (positive), % (freq.)	58.2 (586)	55.0 (275)	61.9 (164)	61.0 (64)	60.6 (83)	0.223
Primary site of infection, % (freq.)						< 0.001
Lung	31.3 (317)	35.0 (177)	31.7 (84)	21.7 (23)	24.1 (33)	
Abdomen	26.8 (271)	22.8 (115)	28.7 (76)	43.4 (46)	24.8 (34)	
Urinary tract	18.6 (188)	20.6 (104)	14.0 (37)	16.0 (17)	21.9 (30)	
Skin/soft tissue	7.7 (98)	8.5 (43)	14.0 (37)	5.7 (6)	8.8 (12)	
Intravenous catheter	1.7 (17)	2.0 (10)	0.8 (2)	2.8 (3)	1.5 (2)	
Bone/joint	1.7 (17)	1.8 (9)	0.8 (2)	0.9 (1)	3.6 (5)	
CNS	1.9 (19)	2.4 (12)	2.3 (6)	0.9 (1)	0 (0)	
Endocardium	1.4 (14)	1.6 (8)	0.8 (2)	0.9 (1)	2.2 (3)	
Implant device	0.6 (6)	0.6 (3)	0 (0)	0.9 (1)	1.5 (2)	
Wound	1.2 (12)	1.2 (6)	1.5 (4)	0.9 (1)	0.7 (1)	
Others	5.3 (54)	3.6 (18)	5.7 (15)	5.7 (6)	10.9 (15)	
Therapeutic interventions						
Mechanical ventilation, % (freq)	42.4 (422)	38.4 (191)	45.8 (120)	54.9 (56)^a^	40.7 (55)	0.026
PMX-DHP, % (freq)	9.1 (89)	7.4 (37)	8.3 (21)^a^	15.6 (15)^a,b^	12.0 (16)	< 0.001
IVIg, % (freq)	20.5 (201)	13.5 (67)	26.1 (66)^a^	30.5 (29)^a^	29.3 (39)^a^	< 0.001
Antithrombin, % (freq)	22.2 (218)	14.3 (71)	25.3 (64)^a^	38.9 (37)^a,b^	32.6 (43)^a^	< 0.001
rTM, % (freq)	30.4 (228)	21.1 (87)	38.3 (70)^a^	61.0 (36)^a,b^	36.8 (35)^a^	< 0.001
Protease inhibitor, % (freq)	8.1 (79)	7.2 (36)	8.7 (22)^a^	11.6 (11)^a^	7.5 (10)^a^	0.001
CRRT, % (freq)	27.7 (271)	22.3 (111)	26.6 (67)^a^	41.7 (40)^a,b^	39.8 (53)^a^	< 0.001
Corticosteroids, % (freq)	28.9 (296)	24.3 (121)	30.8 (78)^a^	40.0 (38)^a,b^	45.0 (59)^a^	< 0.001
Noradrenaline, % (freq)	66.1 (649)	58.7 (294)	70.8 (179)^a^	78.1 (75)^a^	76.5 (101)^a^	< 0.001
Enteral nutrition, % (freq)	49.4 (482)	45.5 (235)	52.2 (131)^a^	45.3 (43)^a^	55.3 (73)^c^	< 0.001

The *P*-values shown in this table were obtained by comparing variables among four groups (PT-INR ≤ 1.2, 1.2 < PT-INR ≤ 1.4, 1.4 < PT-INR ≤ 1.6, and 1.6 < PT-INR) using the Kruskal–Wallis one-way analysis

SOFA, SIRS, and DIC scores in this table represent those at the time of admission

*ADL,* activities of daily living; *APACHE,* Acute Physiology and Chronic Health Evaluation; *CNS,* central nerve system; *CRRT,* continuous renal replacement therapy; *DIC,* disseminated intravascular coagulation; *IVIg,* intravenous immunoglobulin; *JAAM,* Japanese Association for Acute Medicine; *PMX-DHP,* polymyxin B direct hemoperfusion; *PT-INR,* prothrombin time-international normalized ratio; *SIRS,* systemic inflammatory response syndrome; *SOFA,* Sequential Organ Failure Assessment

^a^*P* < 0.05 vs. PT-INR ≤ 1.2

^b^*P* < 0.05 vs. 1.2 < PT-INR ≤ 1.4

^c^*P* < 0.05 vs. 1.4 < PT-INR ≤ 1.6 using the Mann–Whitney U test

**Fig. 1:**
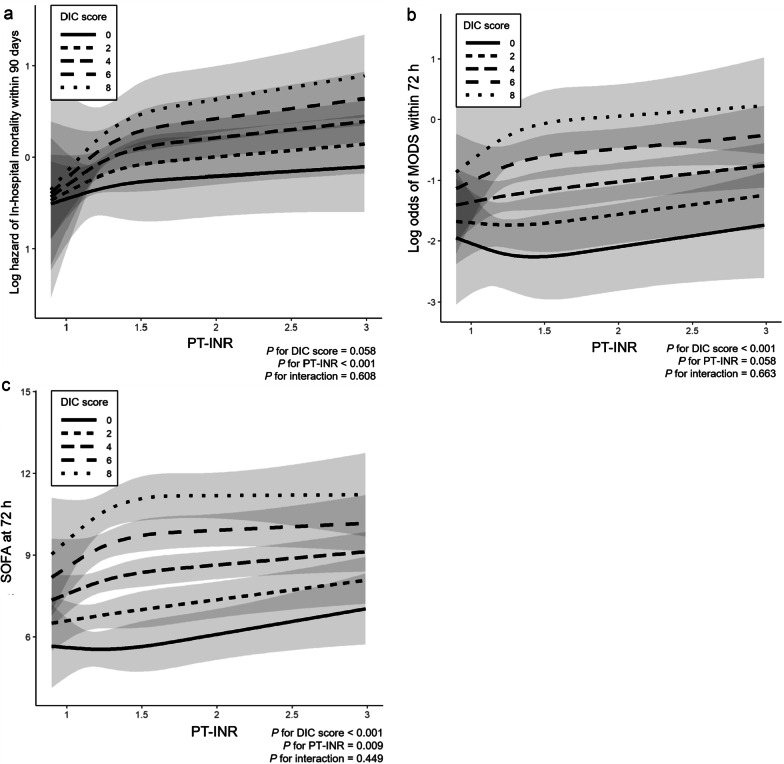
In-hospital mortality within 90 days after admission and organ dysfunction within 72 h after admission. **a** Regression lines of in-hospital mortality estimated by multivariable Cox proportional hazard regression model including the cross-product term between the DIC score and PT-INR value. In-hospital mortality increased with higher values of PT-INR, and this trend was more prominent with higher DIC scores. **b** Regression lines of the development of MODS estimated by multivariate logistic regression analysis. The odds of the development of MODS in patients with DIC increased with higher PT-INR values and DIC scores. **c** Regression lines of the SOFA scores at 72 h after admission estimated by a multivariable linear regression model. The SOFA score was higher with higher PT-INR values, and this trend was more prominent with higher DIC scores. Solid and dashed lines indicate the estimated log-transformed hazard, and shaded areas represent 95% confidence intervals. *DIC,* disseminated intravascular coagulation; *PT-INR,* prothrombin time-international normalized ratio; *MODS,* multiple organ dysfunction syndrome; *SOFA,* Sequential Organ Failure Assessment

Figure 1a shows the results of a multivariable Cox proportional hazard regression model including the cross-product term between the DIC score and PT-INR. For PT-INR values approximately < 1.5, the hazard of in-hospital mortality markedly increased with increasing PT-INR values, and the changes were more pronounced with higher DIC scores. In addition, similar results were found regarding the development of MODS in patients with DIC (DIC score ≥ 4) according to a multivariate logistic regression analysis (Fig. 1b). A multivariable non-linear regression model indicated that the SOFA scores at 72 h after admission increased with higher PT-INR values, and this trend was more prominent with higher DIC scores within the range of less than approximately 1.5 (Fig. 1c).

### Efficacy of anticoagulant therapy according to the DIC score and PT-INR value

**Fig. 2:**
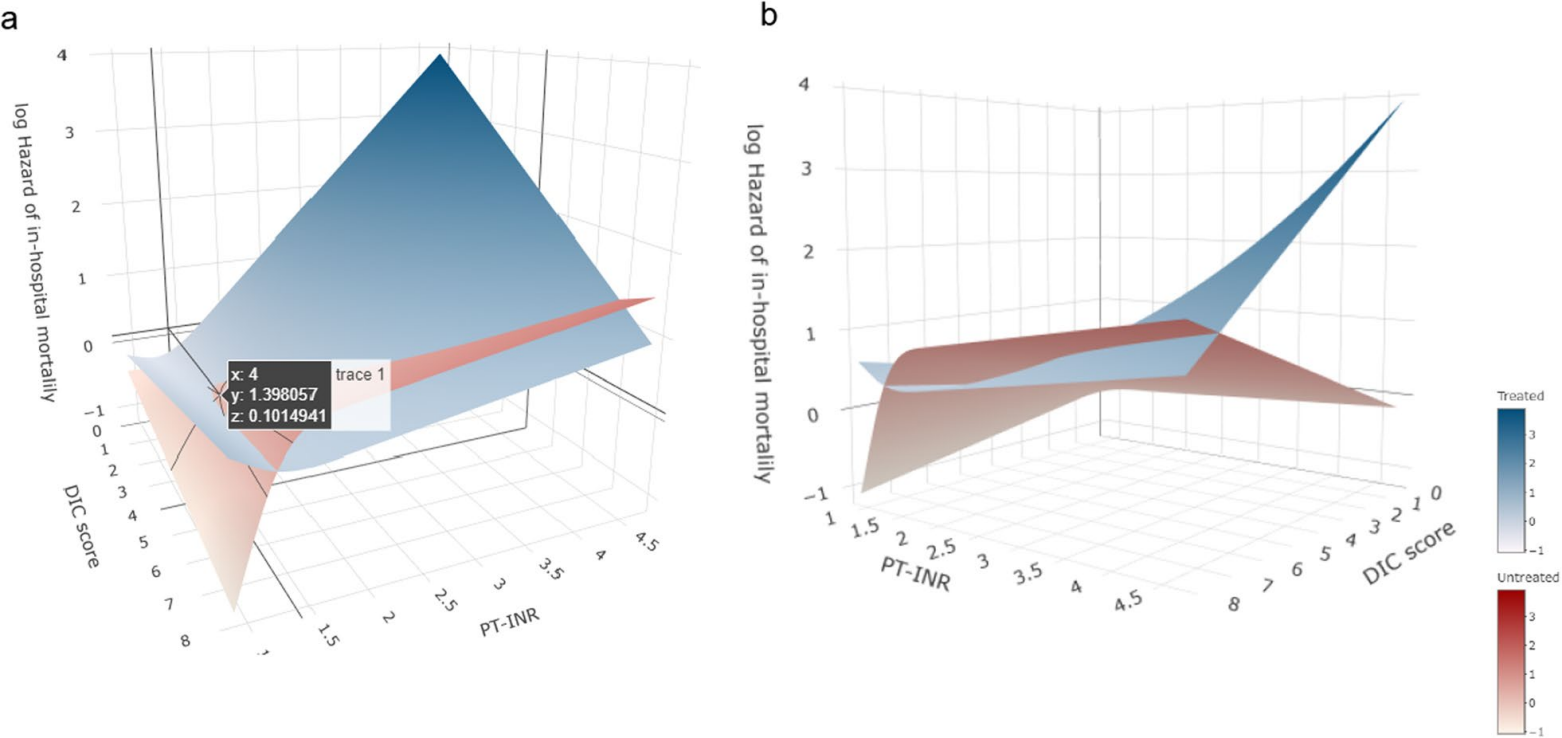
The hazard of in-hospital mortality by PT-INR value and DIC score. **a** When the DIC score is ≥ 4, the hazard of in-hospital mortality is equivalent in patients with and without anticoagulant therapy when the PT-INR value = 1.4. **b** The survival benefit of anticoagulant therapy was confirmed in the patient population with higher DIC scores and PT-INR values. **a**, **b** Are originally similar, shown at different viewing angles to demonstrate the results. The plates represent the estimated log-transformed relative hazard. The blue plate indicates patients who received anticoagulant therapy, and the red plate indicates those who did not. *DIC,* disseminated intravascular coagulation; *PT-INR,* prothrombin time-international normalized ratio

The median doses within 24 and 72 h after admission for patients who received AT were 1500 U (interquartile range, 1500–1500) and 4500 U (interquartile range, 1500–4500), respectively, whereas those of patients who received rhTM were 12,800 U (interquartile range, 6400–19,200) and 30,720 U (interquartile range, 17,160–55,680), respectively. Regardless of the DIC score within the range of ≥ 4, the hazard of in-hospital mortality was equivalent in patients with and without anticoagulant therapy at a PT-INR value of 1.4 (Fig. 2a). A survival benefit from anticoagulant therapy was confirmed in patients with a higher DIC score and PT-INR value (Fig. 2b) (a 3D digital figure that can trace the coordinates and be rotated is shown in Additional file 2: Fig. S2). Notably, anticoagulant therapy in patients with both lower DIC scores and PT-INR values increased the hazard of in-hospital mortality. Based on this result, we compared the disease severity according to the SOFA and APACHE II scores at hospital admission, as well as the in-hospital mortality between patients with and without anticoagulant therapy, to determine the clinical threshold for this treatment. Patients with a DIC score ≥ 5 and PT-INR ≥ 1.5 showed similar disease severity regardless of anticoagulant therapy, while in-hospital mortality was 30.6% and 46.9% (*P* = 0.067) in those with and without anticoagulant therapy, respectively. For factors associated with severe coagulation disorder, such as DIC score ≥ 6 and PT ≥ 1.5 or DIC score ≥ 6 and PT ≥ 1.6, anticoagulant therapy was significantly associated with better survival outcomes (*P* = 0.010 and *P* = 0.014, respectively) (Table 2).

**Table 2:** SOFA score, APACHE II score, and in-hospital mortality in patients groups categorized by values of the DIC score and PT-INR

	Overall	Untreated	Treated	*P*-value
DIC score ≥ 4 and PT-INR ≥ 1.3	*n* = 295	*n* = 139	*n* = 156	
SOFA score 0 h	11 (8–13)	11 (7–12)	11 (8–13)	0.078
APACHE II score	25 (19–32)	23 (17–29)	27 (20–34)	0.003
In-hospital mortality, % (freq.)	28.5% (84)	26.6% (37)	29.7% (47)	0.505
DIC score ≥ 4 and PT-INR ≥ 1.4	*n* = 215	*n* = 99	*n* = 116	
SOFA score 0 h	11 (8–13)	11 (7–12)	11 (8–13)	0.219
APACHE II score	26 (20–33)	26 (17–29)	27 (21–34)	0.108
In-hospital mortality, % (freq.)	32.6% (70)	31.3% (31)	33.6% (39)	0.719
DIC score ≥ 4 and PT-INR ≥ 1.5	*n* = 143	*n* = 62	*n* = 81	
SOFA score 0 h	12 (9–14)	12 (10–14)	11 (7–13)	0.829
APACHE II score	26 (20–33)	26 (19–31)	25 (19–33)	0.961
In-hospital mortality, % (freq.)	35.7% (51)	41.9% (26)	30.9% (25)	0.171
DIC score ≥ 4 and PT-INR ≥ 1.6	*n* = 109	*n* = 49	*n* = 60	
SOFA score 0 h	12 (9–14)	12 (9–13)	11 (8–14)	0.904
APACHE II score	26 (20–33)	26 (18–30)	27 (21–33)	0.943
In-hospital mortality, % (freq.)	42.2% (46)	46.9% (23)	38.3% (23)	0.366
DIC score ≥ 5 and PT-INR ≥ 1.3	*n* = 233	*n* = 102	*n* = 131	
SOFA score 0 h	11 (8–13)	11 (7–13)	11 (8–13)	0.581
APACHE II score	26 (19–33)	26 (17–29)	26 (20–34)	0.136
In-hospital mortality, % (freq.)	31.3% (73)	31.4% (32)	31.3% (41)	0.990
DIC score ≥ 5 and PT-INR ≥ 1.4	*n* = 177	*n* = 76	*n* = 101	
SOFA score 0 h	11 (9–14)	11 (7–12)	11 (8–13)	0.667
APACHE II score	26 (20–33)	26 (17–29)	27 (21–34)	0.431
In-hospital mortality, % (freq.)	35.0% (62)	35.5% (27)	34.5% (35)	0.904
DIC score ≥ 5 and PT-INR ≥ 1.5	*n* = 121	*n* = 49	*n* = 72	
SOFA score 0 h	12 (9–14)	12 (11–14)	11 (8–13)	0.480
APACHE II score	26 (21–33)	28 (21–33)	26 (20–33)	0.616
In-hospital mortality, % (freq.)	37.2% (45)	46.9% (23)	30.6% (22)	0.067
DIC score ≥ 5 and PT-INR ≥ 1.6	*n* = 92	*n* = 38	*n* = 54	
SOFA score 0 h	12 (9–14)	12 (9–14)	11 (10–13)	0.533
APACHE II score	27 (22–33)	26 (18–32)	28 (22–33)	0.474
In-hospital mortality, % (freq.)	43.5% (40)	52.6% (20)	37.0% (20)	0.137
DIC score ≥ 6 and PT-INR ≥ 1.3	*n* = 140	*n* = 58	*n* = 82	
SOFA score 0 h	12 (9–14)	11 (7–14)	12 (9–14)	0.970
APACHE II score	28 (20–34)	25 (17–31)	29 (22–35)	0.239
In-hospital mortality, % (freq.)	31.4% (44)	34.5% (20)	29.3% (24)	0.513
DIC score ≥ 6 and PT-INR ≥ 1.4	*n* = 109	*n* = 45	*n* = 64	
SOFA score 0 h	12 (9–14)	10 (7–14)	12 (9–14)	0.712
APACHE II score	27 (20–33)	21 (15–30)	29 (23–34)	0.332
In-hospital mortality, % (freq.)	32.1% (35)	37.8% (17)	28.1% (18)	0.288
DIC score ≥ 6 and PT-INR ≥ 1.5	*n* = 76	*n* = 30	*n* = 46	
SOFA score 0 h	13 (10–14)	13 (10–14)	12 (10–14)	0.239
APACHE II score	28 (22–33)	29 (20–33)	29 (22–33)	0.601
In-hospital mortality, % (freq.)	32.9% (25)	50.0% (15)	21.7% (10)	0.010
DIC score ≥ 6 and PT-INR ≥ 1.6	*n* = 61	*n* = 25	*n* = 36	
SOFA score 0 h	12 (9–14)	11 (9–14)	12 (9–14)	0.195
APACHE II score	28 (22–33)	28 (18–32)	27 (21–33)	0.154
In-hospital mortality, % (freq.)	41.0% (25)	56.0% (14)	25.0% (9)	0.014

The *P*-values shown in this table were obtained by comparing variables between two groups (treated and untreated) using the Mann–Whitney U test

*APACHE,* Acute Physiology and Chronic Health Evaluation; *DIC,* disseminated intravascular coagulation; *PT-INR,* prothrombin time-international normalized ratio; *SOFA,* Sequential Organ Failure Assessment

**Fig. 3:**
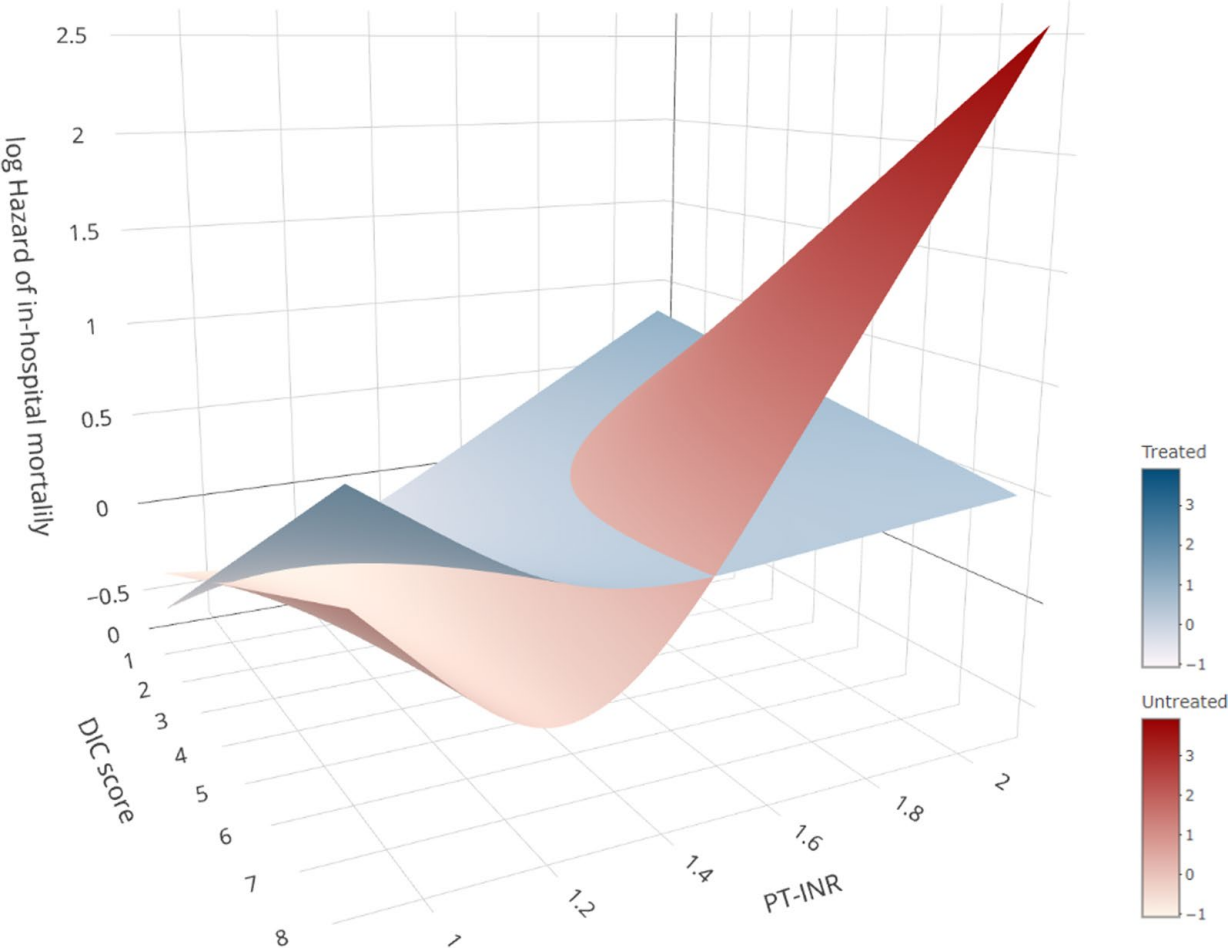
The hazard of in-hospital mortality by PT-INR value and DIC score in patients with PT-INR ≤ 2.2. The survival benefit of anticoagulant therapy was found to be more pronounced with increasing DIC score and PT-INR value. The plates represent the estimated log-transformed relative hazard. The blue plate indicates patients who received anticoagulant therapies, and the red plate indicates those who did not. *DIC,* disseminated intravascular coagulation; *PT-INR,* prothrombin time-international normalized ratio

We then performed the same abovementioned analysis for the cohort of patients with PT-INR values ≤ 2.2 to exclude the effect of an extremely high PT-INR, which could have been due to the prescribed anticoagulants. The survival benefit of anticoagulant therapy was found to be more strongly associated with increased DIC scores and PT-INR values (Fig. 3; Additional file 2: Fig. S3: 3D digital figure).

## Discussion

Our previous study found that anticoagulant therapy was associated with better outcomes according to both DIC and disease severity. Based on these findings, this study assessed the association of in-hospital mortality and organ dysfunction with JAAM DIC score, which is sensitive, simple, and available worldwide, and the PT-INR, a specific surrogate marker of disease severity. We then evaluated the interaction between these two indicators and the impact of anticoagulant therapy on in-hospital mortality. Then, we established a threshold to identify the patient population that would best benefit from this therapy. The degree of organ dysfunction became more severe and in-hospital mortality increased with increasing PT-INR. This trend was more prominent with higher DIC scores. Although patients with anticoagulant therapy in the lower range of DIC scores and PT-INR values had a higher risk of in-hospital mortality, anticoagulant therapy was associated with better survival outcomes in patients with higher DIC scores and PT-INR values. Among those with a DIC score ≥ 5 and PT-INR ≥ 1.5, patients receiving anticoagulant therapy had a lower risk of in-hospital mortality compared with those without anticoagulant therapy.

Recent studies have shown that anticoagulant therapy should be given to patients with a combination of sepsis, DIC, and high disease severity, rather than to those with sepsis only, which has been the target population in most previous large-scale RCTs [27–32]. The SCARLET trial [33], the most recent RCT that evaluated the efficacy of rhTM, adopted sepsis-associated coagulopathy characterized by PT-INR > 1.4, platelet count of 30–150 × 10^9^/L, or a decrease in platelet count > 30% within 24 h, as inclusion criteria. The disease severity in the patient population defined by sepsis-associated coagulopathy was likely to be milder than that defined by DIC, and it was suggested that the enrollment criteria should be modified [34]. In fact, post hoc, sub-analysis of the SCARLET trial showed that patients receiving rhTM with higher levels of thrombin generation biomarkers, including prothrombin fragment 1 + 2 and thrombin antithrombin complex, had lower mortality rates compared with those receiving a placebo [35]. Importantly, this study demonstrated the harmful effects of anticoagulant therapy on sepsis patients without DIC or with lower PT-INR values. This result is supported by the concept of immunothrombosis, a physiological process characterized by the coagulofibrinolytic responses against sepsis, which is not an indication for targets of anticoagulant therapy [3, 36].

Our previous study indicated that anticoagulant therapy can be more effective when based on the level of aggravation of DIC and disease severity as evaluated by the ISTH overt DIC diagnostic criteria and APACHE II score, respectively [37]. As shown in Fig. 2, the issues investigated in the previous study can be evaluated using the DIC score and PT-INR value. Based on these results, we have identified a DIC score ≥ 5 and PT-INR value ≥ 1.5 as the threshold for identifying the optimal target population for anticoagulant therapy. It is important to note that the proportion of patients with PT-INR values > 1.6 receiving AT or rhTM was lower than that of patients with PT-INR values between 1.4 and 1.6, which may be owing to hesitation regarding anticoagulant therapy with AT or rhTM because of concerns about bleeding complications. This study supports a major purpose of diagnostic criteria: to diagnose diseases to improve patient outcomes by intervening with specific treatments [38].

This study had some limitations. First, although the data set was prospectively collected, we were unable to define causal relationships due to the retrospective study design. Second, this study did not assess the efficacy of individual anticoagulant drugs or concomitant therapy because we defined anticoagulant therapy as the administration of rhTM, antithrombin, or their combination. In addition, this study was unable to evaluate the duration and dosage of anticoagulant agents. Third, the efficacy of anticoagulant therapy might not have been evaluated correctly because we did not exclude patients with prescribed anticoagulants as with our previous studies [8, 37, 39]. However, sensitivity analysis of a cohort of patients with a PT-INR value ≤ 2.2 to exclude the effect of patients with an extremely high PT-INR (possibly due to the prescribed anticoagulants) confirmed the robustness of the results regarding the interaction between anticoagulant therapy and in-hospital mortality. Fourth, the data set used in this study did not include several important variables, such as adverse events associated with anticoagulant therapies, including bleeding complications, or the administration of heparin, which is widely used for the prevention of venous thromboembolism, and the administration of coagulation factors corrected prior to ICU admission. Fifth, we estimated regression curves using restricted-cubic-splines, a reasonable choice for assessing the non-linear association between a predictor and an outcome. However, restricted-cubic-spline linearly fits for both ends of the distribution, which do not always provide well-fitted estimates of the actual observations on these regions. Therefore, we should carefully interpret the estimates among the end of the distributions of the predictors. Sixth, data required to control potential confounders might have resulted in biased estimates of the effects. Finally, the study being conducted in a single country may limit the generalizability of the obtained results.

## Conclusion

Organ dysfunction associated with sepsis and in-hospital mortality worsened with higher PT-INR values, and this trend was more prominent with higher DIC scores. Anticoagulant therapy was associated with better survival outcomes based on increases in the DIC scores and PT-INR values, while it should be noted that anticoagulant therapy would be harmful to patients with extremely high PT-INR values, probably caused by the prescribed anticoagulants. Moreover, among patients with a DIC score ≥ 5 and PT-INR value ≥ 1.5, those receiving anticoagulant therapy had lower in-hospital mortality compared with those without anticoagulant therapy. The effects of anticoagulant drugs must be properly assessed in RCTs with robust study designs to further benefit more patients with sepsis. The current results will provide important evidence for designing future RCTs evaluating the effects of anticoagulant therapies for sepsis.

## Supplementary Information

**Additional file 1: Table S1.** Scoring system for DIC according to the JAAM. **Table S2.** Platelet counts, global markers of coagulation, and fibrinolysis at hospital arrival in sepsis patients according to PT-INR value. **Table S3.** Clinical outcomes and PT-INR at hospital arrival.

**Additional file 2: Figure S1.** Flowchart of the study population. **Figure S2.** Original three-dimensional representation shown in Fig. 2. **Figure S3.** Original three-dimensional representation shown in Fig. 3.

## Data Availability

The data that support the findings of this study are available from the authors upon reasonable request.

## Abbreviations

ADL: Activities of daily living
APACHE: Acute Physiology and Chronic Health Evaluation
CCI: Charlson Comorbidity Index
DIC: Disseminated Intravascular Coagulation
FDP: Fibrin/fibrinogen degradation products
FORECAST: Focused Outcomes Research in Emergency Care in Acute Respiratory Distress Syndrome, Sepsis, and Trauma
ICUs: Intensive care units
ISTH: International Society on Thrombosis and Haemostasis
JAAM: Japanese Association for Acute Medicine
J-SSCG 2020: Japanese Clinical Practice Guidelines for Management of Sepsis and Septic Shock 2020
MODS: Multiple organ dysfunction syndrome
PT: Prothrombin time
PT-INR: Prothrombin time-international normalized ratio
qSOFA: Quick Sequential Organ Failure Assessment
RCTs: Randomized controlled trials
rhTM: Recombinant human thrombomodulin
SIRS: Systemic inflammatory response syndrome
SOFA: Sequential Organ Failure Assessment
SSCG: Survival Sepsis Campaign Guidelines for Management of Sepsis and Septic Shock
