# Supplementary material for: Sepsis-related coagulopathy treatment based on the disseminated intravascular coagulation diagnostic criteria: a post-hoc analysis of a prospective multicenter observational study
Source: J Intensive Care. 2023 Mar 5;11:8. doi: 10.1186/s40560-023-00656-5 (PMC9985865; doi:10.1186/s40560-023-00656-5)
Supplement: Supplementary file 1 — Additional file 1: Table S1. Scoring system for DIC according to the JAAM. Table S2. Platelet counts, global markers of coagulation, and fibrinolysis at hospital arrival in sepsis patients according to PT-INR value. Table S3. Clinical outcomes and PT-INR at hospital arrival. [file 40560_2023_656_MOESM1_ESM.docx]

**Table S1.** Scoring system for DIC according to the JAAM

----------------------------------------------------------------------------------------------------------------

**1. Clinical conditions that may be associated with DIC**

1) Sepsis/severe infection (any micro-organism)

2) Trauma/burn/surgery

3) Vascular abnormalities

- large vascular aneurysms

- giant hemangioma

- vasculitis

4) Severe toxic or immunological reactions

- snakebite

- recreational drugs

- transfusion reactions

- transplant rejection

5) Malignancy (except bone marrow suppression)

6) Obstetric calamities

7) Conditions that may be associated with SIRS

- organ destruction (e.g., severe pancreatitis)

- severe hepatic failure

- ischemia/hypoxia/shock

- heat stroke/malignant syndrome

- fat embolism

- rhabdomyolysis

- other

8) Other

-----------------------------------------------------------------------------------------------------------

**2. Clinical conditions that should be carefully ruled out**

A. Thrombocytopenia

1) Dilution and abnormal distribution

Massive blood loss and transfusion, massive infusion

2) Increased platelet destruction

ITP, TTP/HUS, HIT, drugs, viral infection, alloimmune destruction, APS, HELLP, extracorporeal circulation

3) Decreased platelet production

Viral infection, drugs, radiation, nutritional deficiency (vitamin B_12_, folic acid), disorders of hematopoiesis, liver disease, HPS

4) Spurious decrease

EDTA-dependent agglutinins, insufficient anticoagulation of blood samples

5) Other

Hypothermia, artificial devices in the vessel

B. Prolonged prothrombin time

Anticoagulation therapy, anticoagulant in blood samples, vitamin K deficiency, liver cirrhosis, massive blood loss and transfusion

C. Elevated FDP

Thrombosis, hemostasis and wound healing, hematoma, pleural effusion, ascites, anticoagulant in blood samples, antifibrinolytic therapy

D. Other

---------------------------------------------------------------------------------------------------------------

**3. Diagnostic algorithm for SIRS**

1) Temperature > 38 ℃ or < 36 ℃

2) Heart rate > 90 beats/min

3) Respiratory rate > 20 breaths/min or PaCO_2_ < 32 torr (< 4.3 kPa)

4) White blood cell > 12,000 cells/mm^3^, < 4,000 cells/mm^3^, or 10% immature (band) forms

---------------------------------------------------------------------------------------------------------------

**4. Diagnostic algorithm**

Score

SIRS criteria

≥ 3 1

0–2 0

Platelet counts (10^9^/L)

< 80 or > 50% decrease within 24 h 3

≥ 80 < 120 or > 30% decrease within 24 h 1

≥ 120 0

Prothrombin time (value of patient/normal value)

≥ 1.2 1

< 1.2 0

Fibrin/fibrinogen degradation products (mg/L)

≥ 25 3

≥ 10–< 25 1

< 10 0

Diagnosis

Four points or more DIC

-----------------------------------------------------------------------------------------------------------------

*DIC,* disseminated intravascular coagulation; *JAAM,* Japanese Association for Acute Medicine; *SIRS,* systemic inflammatory response syndrome; *ITP,* idiopathic thrombocytopenic purpura; *TTP,* thrombotic thrombocytopenic purpura; *HUS,* hemolytic uremic syndrome; *HIT,* heparin-induced thrombocytopenia; *APS,* antiphospholipid syndrome; *HELLP,* hemolysis, elevated liver enzymes, and low platelet; *HPS,* hemophagocytic syndrome; *EDTA,* ethylenediaminetetraacetic acid; *FDP,* fibrin/fibrinogen degradation products.

The detailed scoring system is described in reference number 16.

**Table S2.** Platelet counts, global markers of coagulation, and fibrinolysis at hospital arrival in sepsis patients according to PT-INR value

|  | Overall | PT-INR ≤ 1.2  (N = 505) | 1.2 < PT-INR ≤ 1.4  (N = 265) | 1.4 < PT-INR ≤ 1.6  (N = 106) | 1.6 < PT-INR  (N = 137) | *P* |
| --- | --- | --- | --- | --- | --- | --- |
| Platelet (10^9^/L) | 142 (88–217) | 167 (105–228) | 124 (85–221)^a^ | 121 (64–188)^a,b^ | 109 (54–168)^a,b^ | < 0.001 |
| PT-INR | 1.3 (1.1–1.4) | 1.1 (1.0–1.2) | 1.3 (1.3–1.4)^a^ | 1.5 (1.5–1.6)^a,b^ | 2..0 (1.8–2.6)^a,b,c^ | < 0.001 |
| APTT | 36.7 (30.9–45.5) | 32.8 (28.7–38.2) | 37.8 (32.0–46.5)^a^ | 44.7 (35.5–52.7)^a,b^ | 51.1 (44.3–72.3)^a,b,c^ | < 0.001 |
| Fibrinogen (g/L) | 4.36 (3.10–5.79) | 4.58 (3.29–5.99) | 4.53 (3.13–6.02) | 3.83 (2.41–5.21)^a,b^ | 3.68 (2.27–5.01)^a,b^ | < 0.001 |
| FDP (mg/L) | 17 .0 (8.9–38.0) | 15.2 (8.6–28.1) | 17.7 (9.1–40.0)^a^ | 23.9 (12.7–77.1)^a,b^ | 17.0 (8.0–55.2)^c^ | < 0.001 |

The results presented in this table were obtained from analyses of a total of 1,013 cases, excluding 165 patients missing DIC scores or PTINR values at day 0.

The *P*-values shown in this table were obtained by comparing variables among four groups (PT-INR ≤ 1.2, 1.2 < PT-INR ≤ 1.4, 1.4 < PT-INR ≤ 1.6, and 1.6 < PT-INR) using the Kruskal-Wallis one-way analysis.

^a^ *P* < 0.05 vs. PT-INR ≤ 1.2, ^b^ *P* < 0.05 vs. 1.2 < PT-INR ≤ 1.4, and ^c^ *P* < 0.05 vs. 1.4 < PT-INR ≤ 1.6 using the Mann-Whitney U test.

*FDP,* fibrin/fibrinogen degradation products; *PT-INR,* prothrombin time-international normalized ratio; *APTT,* activated partial thromboplastin time.

**Table S3.** Clinical outcomes and PT-INR at hospital arrival

|  |  | Overall | PT-INR ≤ 1.2  (N = 505) | 1.2 < PT-INR ≤ 1.4  (N = 265) | 1.4 < PT-INR ≤ 1.6  (N = 106) | 1.6 < PT-INR  (N = 137) | *P* |
| --- | --- | --- | --- | --- | --- | --- | --- |
| SOFA score 72 h after admission |  | 8 (4–11) | 7 (4–10) | 8 (5–11)^a^ | 8 (6–12)^a^ | 9 (6–13)^a,b^ | < 0.001 |
| MODS 72 h, yes/no |  | 159/561 | 62/289 | 40/153 | 21/55^a^ | 36/64^a,b^ | 0.001 |
| In-hospital mortality, n (%) |  | 215 (21.9) | 88 (17.6) | 52 (20.6) | 25 (26.0) | 50 (36.5)^a,b^ | < 0.001 |

The results presented in this table were obtained from analyses of a total of 1,013 cases, excluding 165 patients missing DIC scores or PTINR values at day 0.

The *P*-values shown in this table were obtained by comparing variables among four groups (PT-INR ≤ 1.2, 1.2 < PT-INR ≤ 1.4, 1.4 < PT-INR ≤ 1.6, and 1.6 < PT-INR) using the Kruskal-Wallis one-way analysis.

^a^ *P* < 0.05 vs. PT-INR ≤ 1.2; ^b^ *P* < 0.05 vs. 1.2 < PT-INR ≤ 1.4 using The Mann-Whitney U test.

*MODS,* multiple organ dysfunction syndrome; *PT-INR,* prothrombin time-international normalized ratio; *SOFA,* Sequential Organ Failure Assessment.
