# Supplementary material for: Sepsis-related coagulopathy treatment based on the disseminated intravascular coagulation diagnostic criteria: a post-hoc analysis of a prospective multicenter observational study
Source: J Intensive Care. 2023 Mar 5;11:8. doi: 10.1186/s40560-023-00656-5 (PMC9985865; doi:10.1186/s40560-023-00656-5)
Supplement: Supplementary file 2 — Additional file 2: Figure S1. Flowchart of the study population. Figure S2. Original three-dimensional representation shown in Fig. 2. Figure S3. Original three-dimensional representation shown in Fig. 3. [file 40560_2023_656_MOESM2_ESM.pptx]

## Slide 1
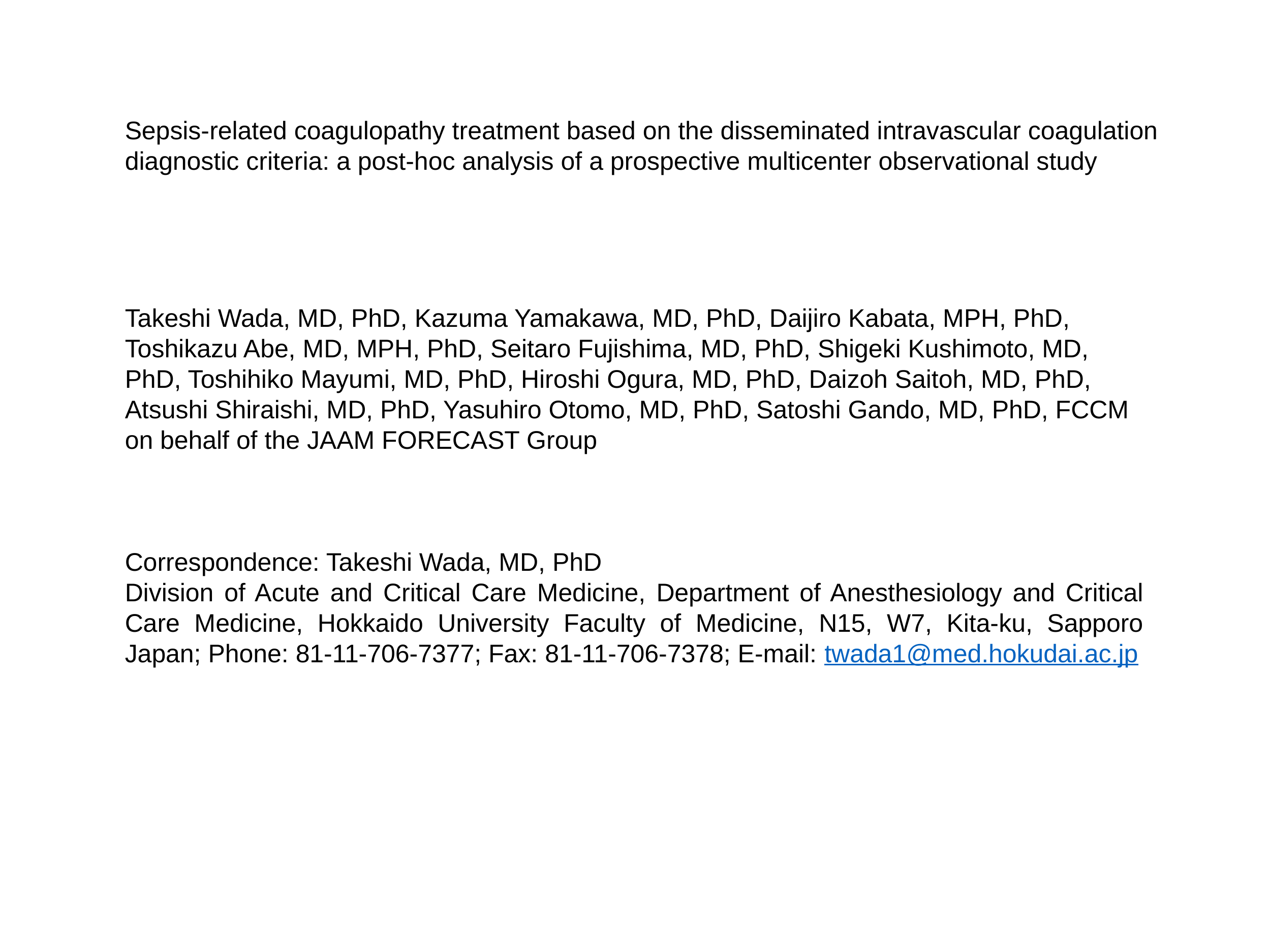

Sepsis-related coagulopathy treatment based on the disseminated intravascular coagulation diagnostic criteria: a post-hoc analysis of a prospective multicenter observational study
Takeshi Wada, MD, PhD, Kazuma Yamakawa, MD, PhD, Daijiro Kabata, MPH, PhD, Toshikazu Abe, MD, MPH, PhD, Seitaro Fujishima, MD, PhD, Shigeki Kushimoto, MD, PhD, Toshihiko Mayumi, MD, PhD, Hiroshi Ogura, MD, PhD, Daizoh Saitoh, MD, PhD, Atsushi Shiraishi, MD, PhD, Yasuhiro Otomo, MD, PhD, Satoshi Gando, MD, PhD, FCCM on behalf of the JAAM FORECAST Group
Correspondence: Takeshi Wada, MD, PhD
Division of Acute and Critical Care Medicine, Department of Anesthesiology and Critical Care Medicine, Hokkaido University Faculty of Medicine, N15, W7, Kita-ku, Sapporo Japan; Phone: 81-11-706-7377; Fax: 81-11-706-7378; E-mail: twada1@med.hokudai.ac.jp

## Slide 2
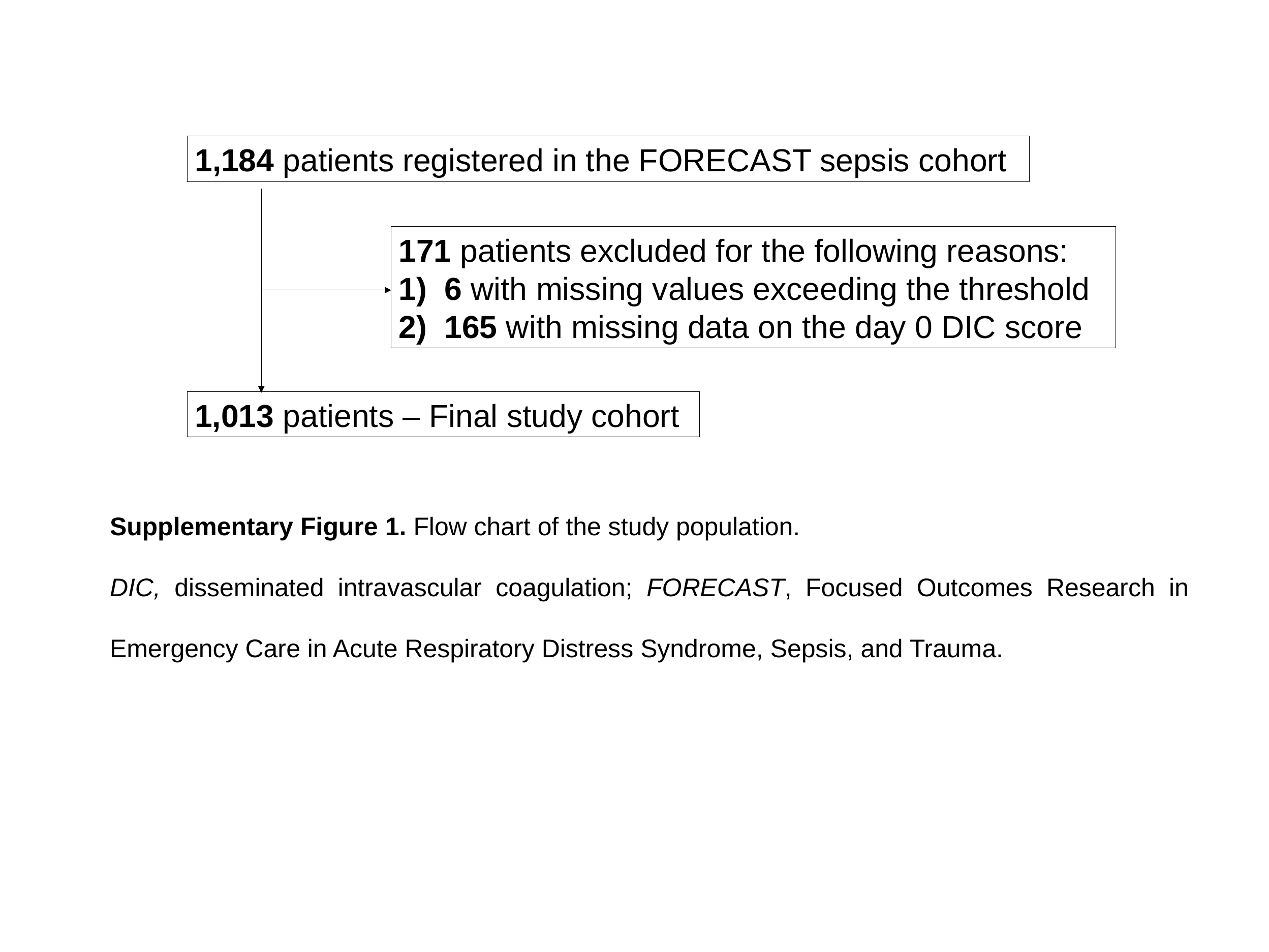

1,184 patients registered in the FORECAST sepsis cohort
171 patients excluded for the following reasons:
1) 6 with missing values exceeding the threshold
2) 165 with missing data on the day 0 DIC score
1,013 patients – Final study cohort
Supplementary Figure 1. Flow chart of the study population.
DIC, disseminated intravascular coagulation; FORECAST, Focused Outcomes Research in Emergency Care in Acute Respiratory Distress Syndrome, Sepsis, and Trauma.

## Slide 3
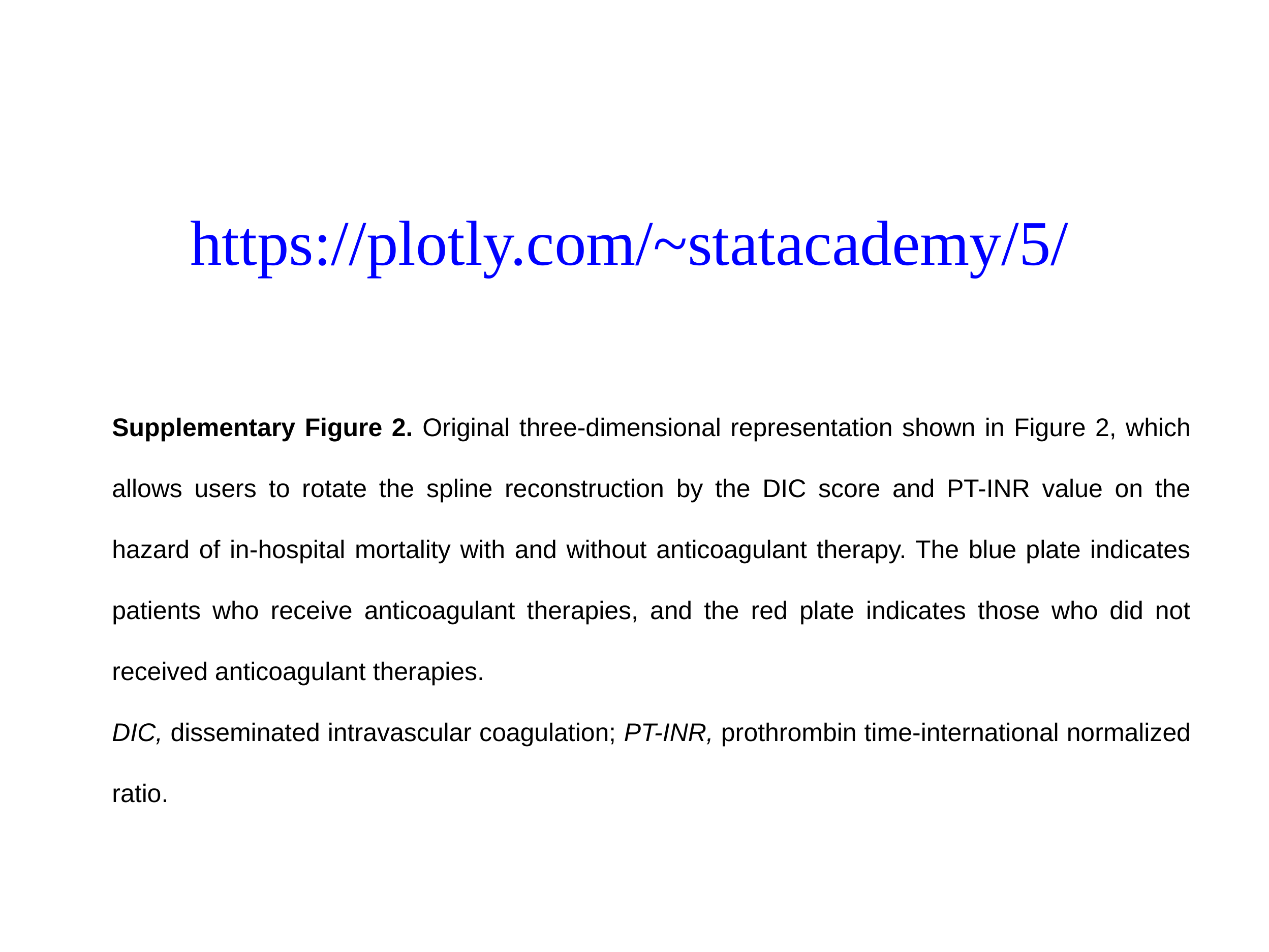

https://plotly.com/~statacademy/5/
Supplementary Figure 2. Original three-dimensional representation shown in Figure 2, which allows users to rotate the spline reconstruction by the DIC score and PT-INR value on the hazard of in-hospital mortality with and without anticoagulant therapy. The blue plate indicates patients who receive anticoagulant therapies, and the red plate indicates those who did not received anticoagulant therapies.
DIC, disseminated intravascular coagulation; PT-INR, prothrombin time-international normalized ratio.

## Slide 4
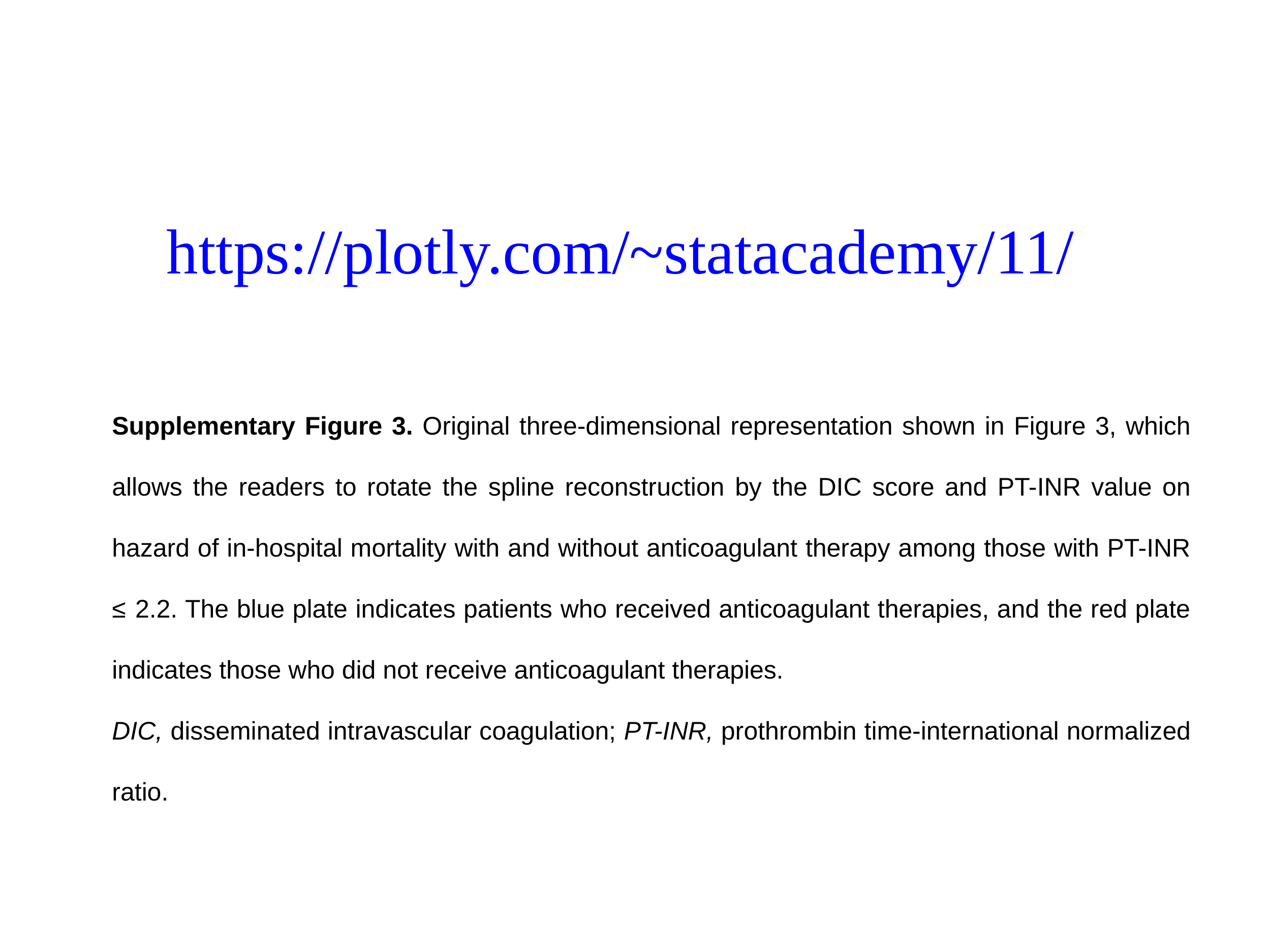

https://plotly.com/~statacademy/11/
Supplementary Figure 3. Original three-dimensional representation shown in Figure 3, which allows the readers to rotate the spline reconstruction by the DIC score and PT-INR value on hazard of in-hospital mortality with and without anticoagulant therapy among those with PT-INR ≤ 2.2. The blue plate indicates patients who received anticoagulant therapies, and the red plate indicates those who did not receive anticoagulant therapies.
DIC, disseminated intravascular coagulation; PT-INR, prothrombin time-international normalized ratio.
